# Design and fabrication of a GaN HEMT power amplifier based on hidden Markov model for wireless applications

**DOI:** 10.1371/journal.pone.0285186

**Published:** 2023-05-05

**Authors:** Mohammad Soruri, S. Mohammad Razavi, Mehdi Forouzanfar, Paolo Colantonio

**Affiliations:** 1 Faculty of Electrical and Computer Engineering, University of Birjand, Birjand, Iran; 2 Electronic Engineering Department, University of Roma Tor Vergata, Roma, Italy; Edinburgh Napier University, UNITED KINGDOM

## Abstract

Improvement of power amplifier’s performance is the desired topic in communication systems. There are many efforts are made to provide good input and output matching, high efficiency, sufficient power gain and appropriate output power. This paper presents a power amplifier with optimized input and output matching networks. In the proposed approach, a new structure of the Hidden Markov Model with 20 hidden states is used for modeling the power amplifier. The widths and lengths of the microstrip lines in the input and output matching networks are defined as the parameters that the Hidden Markov Model should optimize. For validating our algorithm, a power amplifier has been realized based on a 10W GaN HEMT with part number CG2H40010F from the Cree corporation. Measurement results have shown a PAE higher than 50%, a Gain of about 14 dB, and input and output return losses lower than -10 dB over the frequency range of 1.8–2.5 GHz. The proposed PA can be used in wireless applications such as radar systems.

## 1. Introduction

One of the essential components in the structure of a transmitter system is the Power Amplifier (PA) [1]. Since the power amplifier is located at the final stage of the transmitter, its efficiency influences the system’s overall efficiency. PAs are found in the realization of the many microwaves and millimeter-wave systems including radar and antenna systems [2–8], cellular phones [9–11], electronic warfare [12, 13], heating [14, 15], and also many other applications that highlight the importance of such component. Due to the wide variety of PA applications, from wireless communication handsets to heating and electronic warfare, PA is designed and biased in a suitable class to satisfy the desired parameters.

As the input signal of a PA is large, it typically operates in nonlinear conditions and the output signal has some unavoidable distortions. On the other hand, the biasing of a PA also affects its non-linear behavior. Generally, class A or class B structures achieve high linearity. However, for obtaining high linearity, PA must be operated in a low-efficiency region and vice versa [16–18].

For improving the efficiency of a PA, several techniques are proposed [19]. Using switching power amplifiers and Doherty power amplifiers [20] are two traditional techniques for achieving this goal. The Envelope Tracking (ET) technique improves efficiency by adjusting the supply voltage [21]. Also for improving the linearity and efficiency simultaneously, the outphasing technique can be used [22].

In addition, to apply conventional methods to increase the efficiency of a circuit, the use of innovative and evolutionary methods for optimizing discrete and Monolithic Microwave Integrated Circuits (MMIC) has also become common. Evolutionary algorithms and multi-objective optimization were considered in [23]. Power amplifier optimization based on a nonlinear programming technique was studied in [24]. Particle Swarm Optimization (PSO) was used for the optimization of various PAs in [16, 25–27]. Also, Artificial Bee Colony (ABC) and PSO algorithms were applied by Bipin and Rao for the linearization of a PA in [28]. Bayesian optimization for designing broadband and high-efficiency PA was studied in [29], that the proposed algorithm optimized the drain waveforms by maximizing the fundamental output power over the frequency range of 1.5 to 2.5 GHz. Improving the efficiency and gain with automated deep neural learning was obtained over the frequency range of 1.8–2.2 GHz by Koushalvandi et. all in [30].

In this paper, Hidden Markov Model (HMM) is used for improving the parameters of a PA. HMM was first introduced by Baum and Petrie in 1960 [31, 32]. HMM is a statistical model that was used to predict events and model the sequences [33, 34]. In the proposed approach, the widths and lengths of micro-strips are optimized by HMM. HMM is a robust algorithm for simulating the sequences and comparing them with various lengths. It can be considered as a machine with hidden states whose transition among them causes generating the observable states [35]. In the proposed approach for improving the efficiency, HMM is used for modeling the PA and predicting an optimized sentence that includes the widths and lengths of the microstrips in the RF paths.

The paper is organized as follows: In Sect. 2, the overall structure of HMM is discussed. In Sect. 3, the proposed method and its application in the high-efficiency PA are explained. Measurement results and discussion are presented in Sect. 4 and finally, a conclusion about the proposed approach is presented in Sect. 5.

## 2. Hidden Markov model

**Fig 1:**
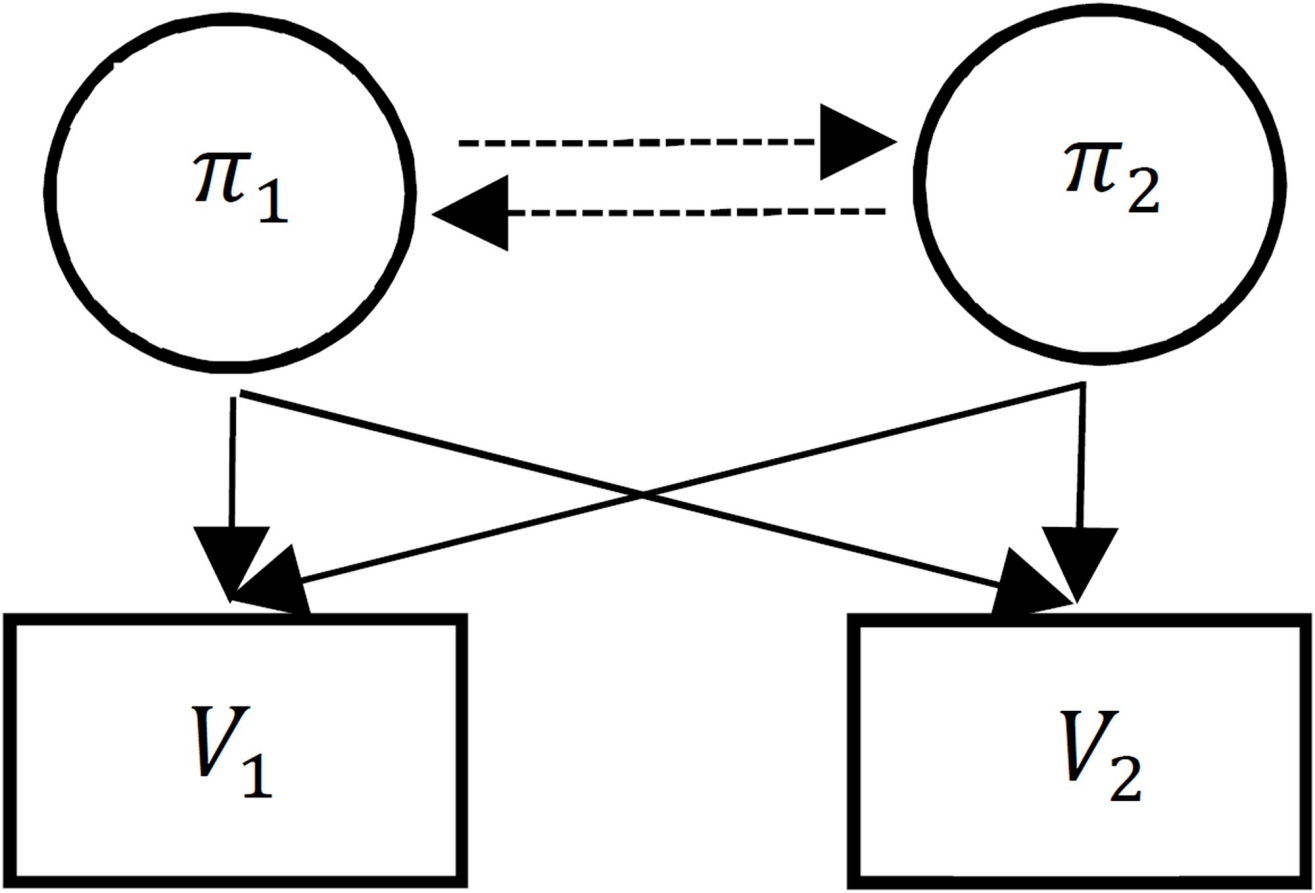
A simple model of HMM, hidden states, and observable states are shown with circles and squares, respectively. Transition probabilities are shown with dashed lines and emission probabilities are shown with solid lines.

HMM consists of several hidden states and several observable states. The transitions between hidden states are determined through probability functions represented by matrix elements {*a*_*ij*_} [36, 37]. Fig 1 represents a simple example of HMM that contains two hidden states (*π*_1_ and *π*_2_), and two observable states (*V*_1_ and *V*_2_). In this structure, each of the two hidden states can emit two observable states.

If we have an HMM with *n* hidden states and *m* observables states, we can use the following equations for modeling the behavior of HMM [37].

$$\pi =\left\{\pi_{1},\pi_{2},\ldots \pi_{n}\right\}$$ (1)

$$a_{ij}=P\left(\pi_{j}\left(t+1\right)|\pi_{i}\left(t\right)\right)\;\;1<i,j<n$$ (2)

$$\overset{n}{\underset{J=1}{\sum }}a_{fj}=1,\;1<f<n$$ (3)

Where in Eq 1, π is the set of hidden states, and in Eq 2 *a*_*ij*_ is the transition probability of going from a hidden state *π*_*i*_ to a hidden state *π*_*j*_. Eq 3 states that the sum of the transition probabilities taken out of each hidden state is equal to one. In each hidden state *π*_*i*_, the observable states can be produced [37]. These probabilities are named with emission probabilities that are shown with the elements of matrix *B* = {*b*_*jk*_}.

$$v=\left\{v_{1},v_{2},\ldots v_{m}\right\}$$ (4)

$$b_{jk}=P\left(v_{k}\left(t\right)|\pi_{j}\left(t\right)\right)\;1<j<n,\;1<k<m$$ (5)

$$\overset{m}{\underset{k=1}{\sum }}b_{fk}=1,\;1<f<n$$ (6)

Where in Eq 4, *v* is the set of observable states, and in Eq 5, *b*_*jk*_ is the probability of emitting an observable state *v*_*k*_ in the hidden state *π*_*j*_. Eq 6 states that the sum of the emission probabilities taken out of each hidden state is equal to one. Initial probabilities determine the starting model with each hidden state at the finish. Eq 7 describes the initial probabilities.

$$\pi_{i}=P\left(\pi \left(1\right)=\pi_{i}\left(1\right)\right),\;1<i,j<n$$ (7)

Where in Eq 7, *π*_*i*_ is the probability of starting the model with a hidden state *π*(1). As a result, each HMM is summarized with triple *λ* = (*A*, *B*, *π*) that can model the optimized sequence of widths and lengths of microstrip lines used in the PA matching network.

## 3. High-efficiency PA with HMM algorithm

As said, HMM is a robust algorithm for simulating the sequences, so in the proposed PA, we used HMM for modeling the PA and predicting an optimized sentence that includes the widths and lengths of the microstrips in the RF paths. For using HMM, we should first determine the set of hidden states and transition probabilities matrix and the set of observable states and emission probabilities matrix. After determining the overall structure of HMM, we define the sequence that should be optimized from the HMM structure. This sequence is the widths and lengths of the microstrips line in the RF path. Eq 8 shows this sequence.


$$p=\left[W_{i_{1}},L_{i_{1}},W_{i_{2}},L_{i_{2}},W_{i_{3}},L_{i_{3}},W_{i_{4}},L_{i_{4}},\;W_{d_{1}},L_{d_{1}},W_{d_{2}},L_{d_{2}},W_{o_{1}},L_{o_{1}},W_{o_{2}},L_{o_{2}},W_{o_{3}},L_{o_{3}},W_{o_{4}},L_{o_{4}}\right]$$ (8)

The number of parameters in Eq 8 is proportional to the number of the microstrips lines in the input and output matching networks. In the proposed PA, 4 lines in the input and 6 lines in the output matching network were used to realize wideband matching. The number of lines for matching the input and output can be increased by the cost of increasing the total area of PA. The proposed matching circuit provides a suitable bandwidth, high efficiency, and a suitable gain while occupying a relatively small area.

**Fig 2:**
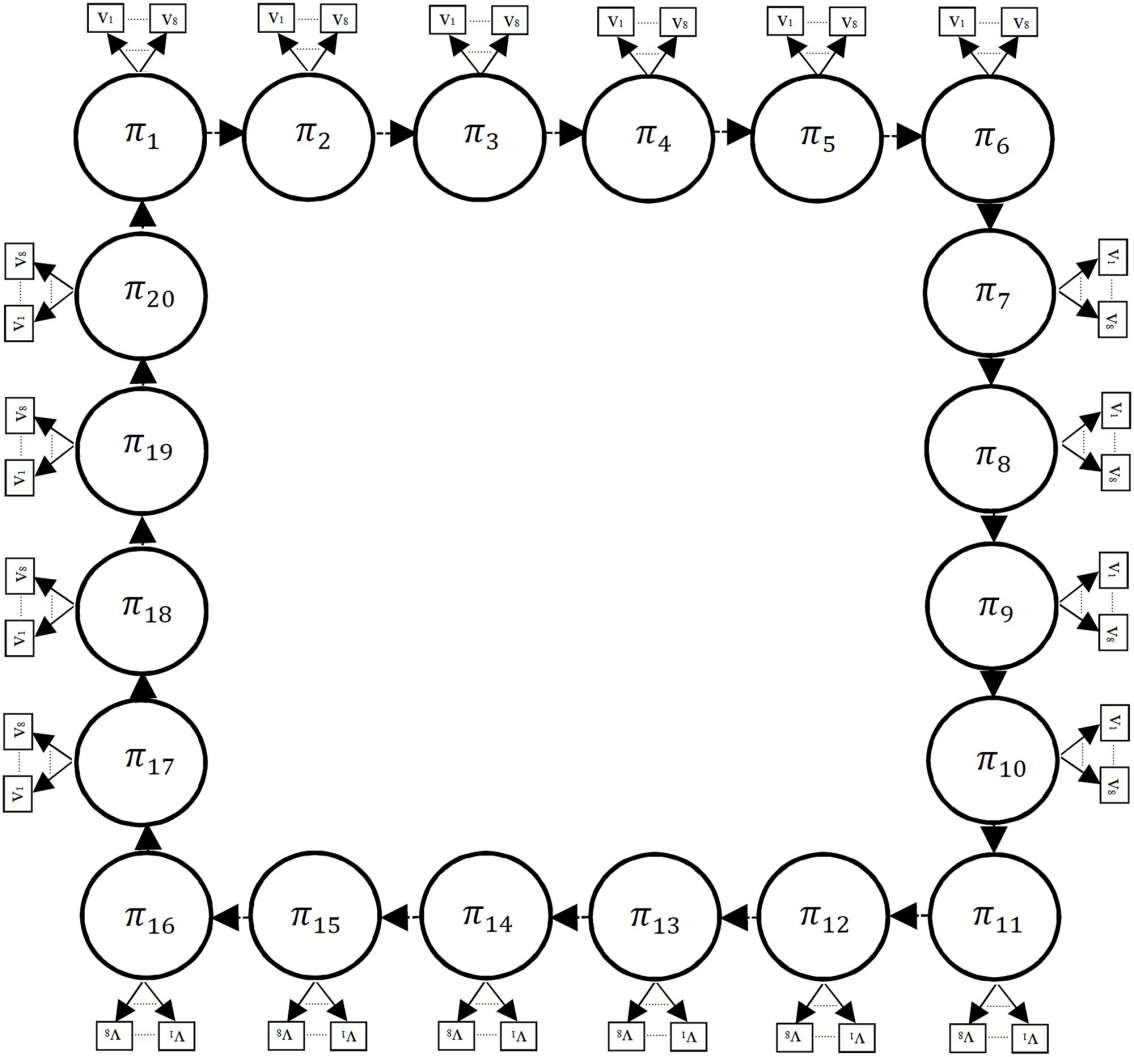
The proposed structure of HMM for generating the optimum parameters of PA.

The proposed structure of HMM is illustrated in Fig 2.

As shown in Fig 2, we considered 20 hidden states for the proposed HMM for modeling 20 parameters in the sequence *p* described in Eq 8. Each hidden state is the length or width of each microstrip that should be optimized and can generate eight observable states. These eight observable values for each hidden state, are close to the initial values obtained from the load-pull analysis and the design of the corresponding matching networks taking into account the initial tunes.

The proposed structure of Fig 2, actually expresses a sequence of the microstrip line values of the power amplifier, which according to the sequence values, the power amplifier can have its best performance. Based on the proposed structure in Fig 2, the transition probabilities matrix, *A*, can be shown in Eq 9.


$$A={\left[\begin{array}{llllllllllllllllllll} 0 & 1 & 0 & 0 & 0 & 0 & 0 & 0 & 0 & 0 & 0 & 0 & 0 & 0 & 0 & 0 & 0 & 0 & 0 & 0\\ 0 & 0 & 1 & 0 & 0 & 0 & 0 & 0 & 0 & 0 & 0 & 0 & 0 & 0 & 0 & 0 & 0 & 0 & 0 & 0\\ 0 & 0 & 0 & 1 & 0 & 0 & 0 & 0 & 0 & 0 & 0 & 0 & 0 & 0 & 0 & 0 & 0 & 0 & 0 & 0\\ 0 & 0 & 0 & 0 & 1 & 0 & 0 & 0 & 0 & 0 & 0 & 0 & 0 & 0 & 0 & 0 & 0 & 0 & 0 & 0\\ 0 & 0 & 0 & 0 & 0 & 1 & 0 & 0 & 0 & 0 & 0 & 0 & 0 & 0 & 0 & 0 & 0 & 0 & 0 & 0\\ 0 & 0 & 0 & 0 & 0 & 0 & 1 & 0 & 0 & 0 & 0 & 0 & 0 & 0 & 0 & 0 & 0 & 0 & 0 & 0\\ 0 & 0 & 0 & 0 & 0 & 0 & 0 & 1 & 0 & 0 & 0 & 0 & 0 & 0 & 0 & 0 & 0 & 0 & 0 & 0\\ 0 & 0 & 0 & 0 & 0 & 0 & 0 & 0 & 1 & 0 & 0 & 0 & 0 & 0 & 0 & 0 & 0 & 0 & 0 & 0\\ 0 & 0 & 0 & 0 & 0 & 0 & 0 & 0 & 0 & 1 & 0 & 0 & 0 & 0 & 0 & 0 & 0 & 0 & 0 & 0\\ 0 & 0 & 0 & 0 & 0 & 0 & 0 & 0 & 0 & 0 & 1 & 0 & 0 & 0 & 0 & 0 & 0 & 0 & 0 & 0\\ 0 & 0 & 0 & 0 & 0 & 0 & 0 & 0 & 0 & 0 & 0 & 1 & 0 & 0 & 0 & 0 & 0 & 0 & 0 & 0\\ 0 & 0 & 0 & 0 & 0 & 0 & 0 & 0 & 0 & 0 & 0 & 0 & 1 & 0 & 0 & 0 & 0 & 0 & 0 & 0\\ 0 & 0 & 0 & 0 & 0 & 0 & 0 & 0 & 0 & 0 & 0 & 0 & 0 & 1 & 0 & 0 & 0 & 0 & 0 & 0\\ 0 & 0 & 0 & 0 & 0 & 0 & 0 & 0 & 0 & 0 & 0 & 0 & 0 & 0 & 1 & 0 & 0 & 0 & 0 & 0\\ 0 & 0 & 0 & 0 & 0 & 0 & 0 & 0 & 0 & 0 & 0 & 0 & 0 & 0 & 0 & 1 & 0 & 0 & 0 & 0\\ 0 & 0 & 0 & 0 & 0 & 0 & 0 & 0 & 0 & 0 & 0 & 0 & 0 & 0 & 0 & 0 & 1 & 0 & 0 & 0\\ 0 & 0 & 0 & 0 & 0 & 0 & 0 & 0 & 0 & 0 & 0 & 0 & 0 & 0 & 0 & 0 & 0 & 1 & 0 & 0\\ 0 & 0 & 0 & 0 & 0 & 0 & 0 & 0 & 0 & 0 & 0 & 0 & 0 & 0 & 0 & 0 & 0 & 0 & 1 & 0\\ 0 & 0 & 0 & 0 & 0 & 0 & 0 & 0 & 0 & 0 & 0 & 0 & 0 & 0 & 0 & 0 & 0 & 0 & 0 & 1\\ 1 & 0 & 0 & 0 & 0 & 0 & 0 & 0 & 0 & 0 & 0 & 0 & 0 & 0 & 0 & 0 & 0 & 0 & 0 & 0 \end{array}\right]}_{20\times 20}$$ (9)

$$\overset{20}{\underset{J=1}{\sum }}a_{fj}=1,\;1<f<20$$ (10)

As is shown in Eq 9 and Fig 2, in transition probability matrix *A*, the probability of going from each hidden state to other hidden states is equal to 1. Eq 11 shows the initial optimized values of microstrip lines that are obtained based on the initial tunes of PA from the load-pull simulation and optimization of the input and output matching network. (All values are in millimeters).


$$p^{*}=\left[14,\;4.5,\;0.6,\;3.2,\;5,\;4.2,\;4.1,\;2.9,\;1.8,\;3.5,\;18.8,\;20.3,\;27.2,\;0.4,\;8.5,9.2,\;5.5,\;1.8,\;7,\;3.2\right]$$ (11)

The set of observable states is determined based on the initial values vectors, p*, in Eq 11. Therefore, for obtaining the emission probabilities matrix, *B*, at first, we should select eight values (observable states) for each of the initial values (hidden states) in Eq 8. These eight observable values are selected according to the initial values of each of the micro-strips values obtained in Eq 11. The emission probabilities matrix, *B*, is expressed by Eq 12.


$$B={\left[\begin{array}{cccc} b_{1,1} & \cdot & \cdot & b_{1,8}\\ \cdot & & & \\ \cdot & & & \\ b_{20,1} & \cdot & \cdot & b_{20,8} \end{array}\right]}_{20\times 8}$$ (12)

$$\overset{8}{\underset{k=1}{\sum }}b_{fk}=1,\;1<f<20$$ (13)

The training of HMM is performed with the Baum-Welch algorithm which is a traditional algorithm for the training of HMM [37]. Eq 13 is the condition related to the sum of emission probabilities taken out of each hidden state, controlled in each training algorithm iteration. All the eight observable values that 20 hidden states can generate are shown in Eq 14 (All values are in millimeters).


$$V={\left[\begin{array}{cccccccc} 13 & 13.3 & 13.7 & 14 & 14.3 & 14.5 & 14.8 & 15.2\\ 3.5 & 3.7 & 4.1 & 4.3 & 4.5 & 4.8 & 5 & 5.2\\ 0.1 & 0.2 & 0.4 & 0.6 & 0.7 & 0.8 & 1 & 1.1\\ 2.4 & 2.6 & 2.8 & 3 & 3.2 & 3.4 & 3.6 & 3.8\\ 4.1 & 4.3 & 4.5 & 4.7 & 5 & 5.3 & 5.6 & 5.8\\ 3.4 & 3.6 & 3.8 & 4 & 4.2 & 4.4 & 4.5 & 4.7\\ 3.5 & 3.7 & 3.9 & 4.1 & 4.3 & 4.5 & 4.8 & 5.1\\ 2.3 & 2.5 & 2.7 & 2.9 & 3.2 & 3.4 & 3.6 & 3.8\\ 1.2 & 1.3 & 1.4 & 1.6 & 1.8 & 2 & 2.2 & 2.4\\ 2.9 & 3.1 & 3.2 & 3.3 & 3.5 & 3.7 & 3.9 & 4.1\\ 17.4 & 17.7 & 18.3 & 18.8 & 19.2 & 19.7 & 19.9 & 20.2\\ 18.3 & 18.7 & 19.2 & 19.8 & 20.3 & 20.8 & 21.4 & 21.9\\ 21.5 & 23 & 25 & 27.2 & 29.5 & 32 & 35 & 37\\ 0.2 & 0.27 & 0.3 & 0.35 & 0.4 & 0.52 & 0.6 & 0.7\\ 6.9 & 7.3 & 7.7 & 8.2 & 8.5 & 8.8 & 9.1 & 9.4\\ 8 & 8.4 & 8.8 & 9.2 & 9.6 & 10 & 10.4 & 10.9\\ 4.1 & 4.3 & 4.6 & 5.1 & 5.5 & 5.8 & 6.1 & 6.4\\ 0.8 & 1.15 & 1.42 & 1.6 & 1.8 & 2 & 2.2 & 2.5\\ 5.2 & 5.5 & 6.1 & 6.4 & 7 & 7.45 & 7.95 & 8.4\\ 2.55 & 2.75 & 2.95 & 3.2 & 3.4 & 3.65 & 4 & 4.25 \end{array}\right]}_{20\times 8}$$ (14)

In the Baum-Welch algorithm, the Parameters of HMM, are obtained based on the training sequences. The number of training sequences is 160 specified based on Eq 14 (160 = 20*8). For training of HMM, the maximum likelihood concept is used which is defined by Eq 15 [37]:

$$F=P\left(S|\lambda \right)$$ (15)

Where in Eq 15, F is the maximum posterior probability of generating sequence S by *λ* = (*A*, *B*, *π*).

We want to find the sequence of observable states that optimize the PA performance. For obtaining the solution, first, we should define a fitness function. In other words, we want to find that sequence of observable states that minimize our fitness function. The fitness function that we define, is the PAE of PA. Eq 16, defines this fitness function [38].

$$PAE=\frac{P_{o}-P_{in}}{P_{DC}}$$ (16)

Where in Eq 16, *P*_*DC*_ is the supply power, and *P*_*in*_ and *P*_*o*_ are the input and output power, respectively.

We can consider the *P*_*DC*_ as the following equation [1].


$$P_{DC}=P_{diss}+P_{out,f}+\overset{\infty }{\underset{n=2}{\sum }}P_{out,nf}$$ (17)

In Eq 17, *P*_*diss*_ is the dissipation power in PA and *P*_*out*,*f*_ and $\Sigma_{n=2}^{\infty }\;P_{out,nf}$ are the fundamental output power and the sum of output powers in the harmonic frequencies, respectively. At the finish, we can state the PAE by Eq 18.


$$PAE=\frac{P_{out,f}-P_{in}}{P_{diss}+P_{out,f}+\sum_{n=2}^{\infty }P_{out,nf}}$$ (18)

In Eq 18, for optimizing the PAE, we should minimize dissipation power and the sum of output power in the harmonic frequencies. It means for obtaining a maximum PAE, this equation should be minimized:

$$P_{diss}+\overset{\infty }{\underset{n=2}{\sum }}P_{out,nf}$$ (19)

So, Eq 19 is the primary cost function for optimizing the HMM. The algorithm is terminated when the maximum number of iterations is reached or the specified error is below a given threshold value.

## 4. Measurement results and discussion

**Fig 3:**
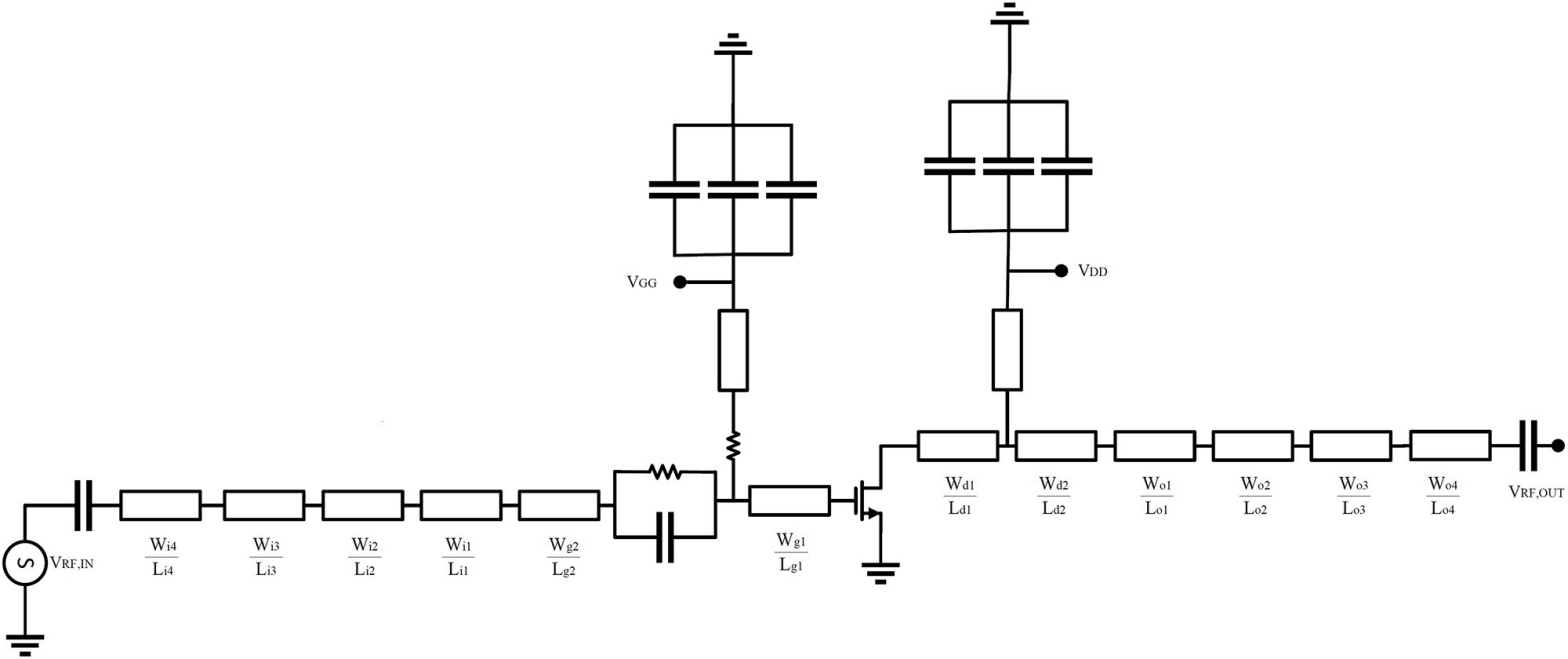
Schematics of the proposed PA.

**Fig 4:**
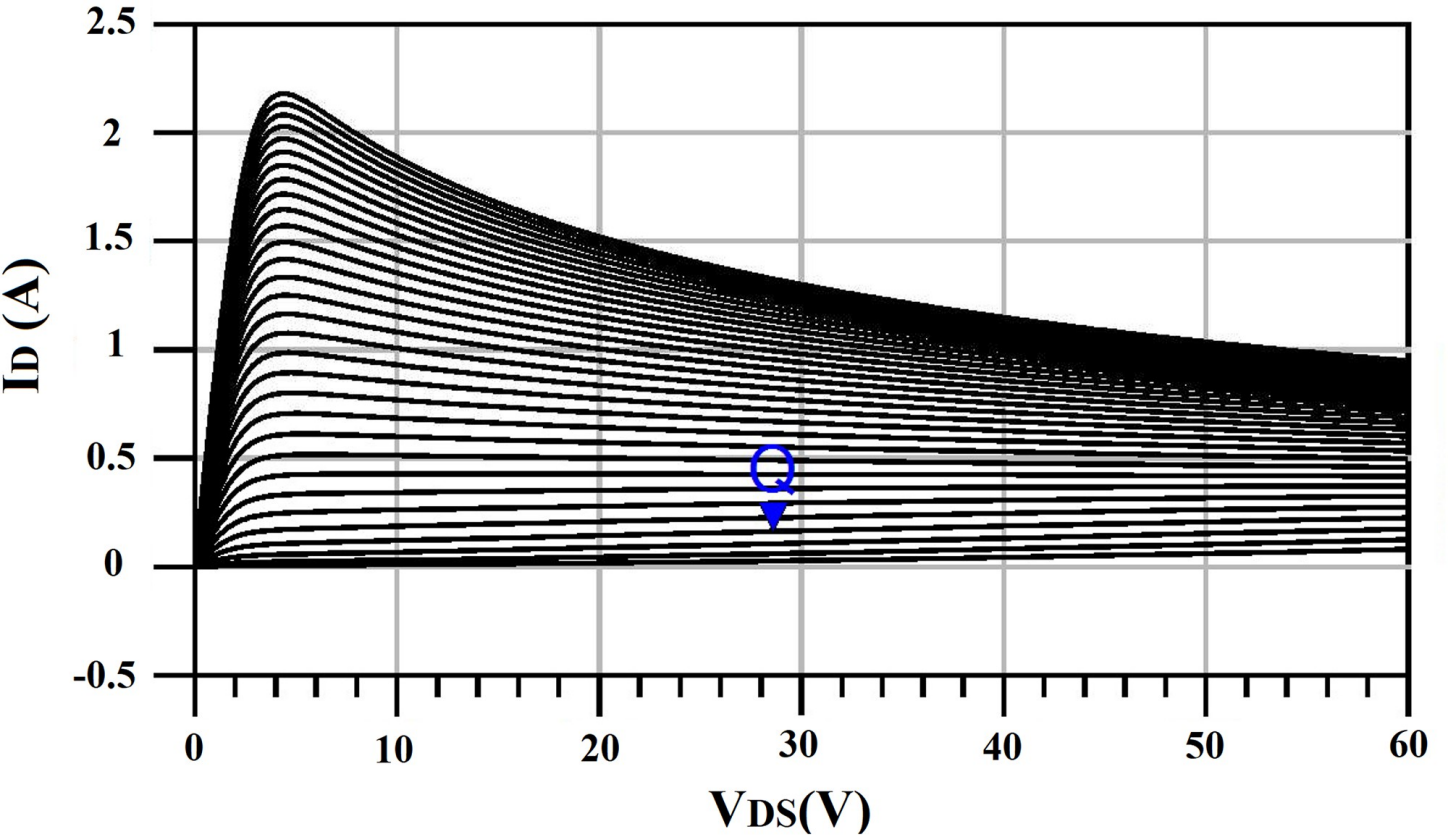
The I-V curve of the transistor, *Q* is the DC bias point.

The proposed algorithm used the ADS and Matlab software for the implementation of the Harmonic Balance of PA and HMM, respectively and during the optimization algorithm, ADS is linked to Matlab [39]. For the realization of PA, we use a GaN HEMT with the part number CGH40010F, from the Cree corporation. The substrate specifications are Rogers 4003, with a thickness of 32 mils and *ε*_*r*_ = 3.55. It should be noted that a deep AB class is selected for biasing of the transistor. The bias voltage of the drain, *V*_*DD*_ is 28V, the bias voltage of the gate, *V*_*GG*_ is -2.74 V, and the drain current, *I*_*D*_ is 160 mA. Fig 3 shows the overall structure of PA and Fig 4 shows the I-V curve of the transistor.

In Fig 3, each line in the RF path of PA is specified by its width and length. For example, the line specified with $\frac{W_{g1}}{L_{g1}}$, is a line with the width of *W*_*g*1_ and length of *L*_*g*1_. As shown in Fig 3, we considered 4 lines in the input and 6 lines in the output matching networks. It should be noted that all high-frequency simulations of the proposed PA were performed in the momentum microwave environment of ADS software using the precise non-linear model of the transistor, which is provided by Cree company.

**Fig 5:**
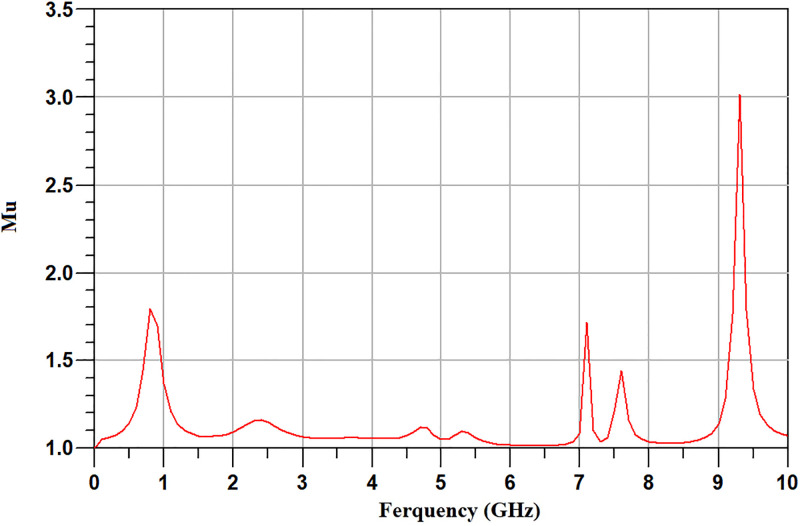
The stability factor of the optimized PA.

**Fig 6:**
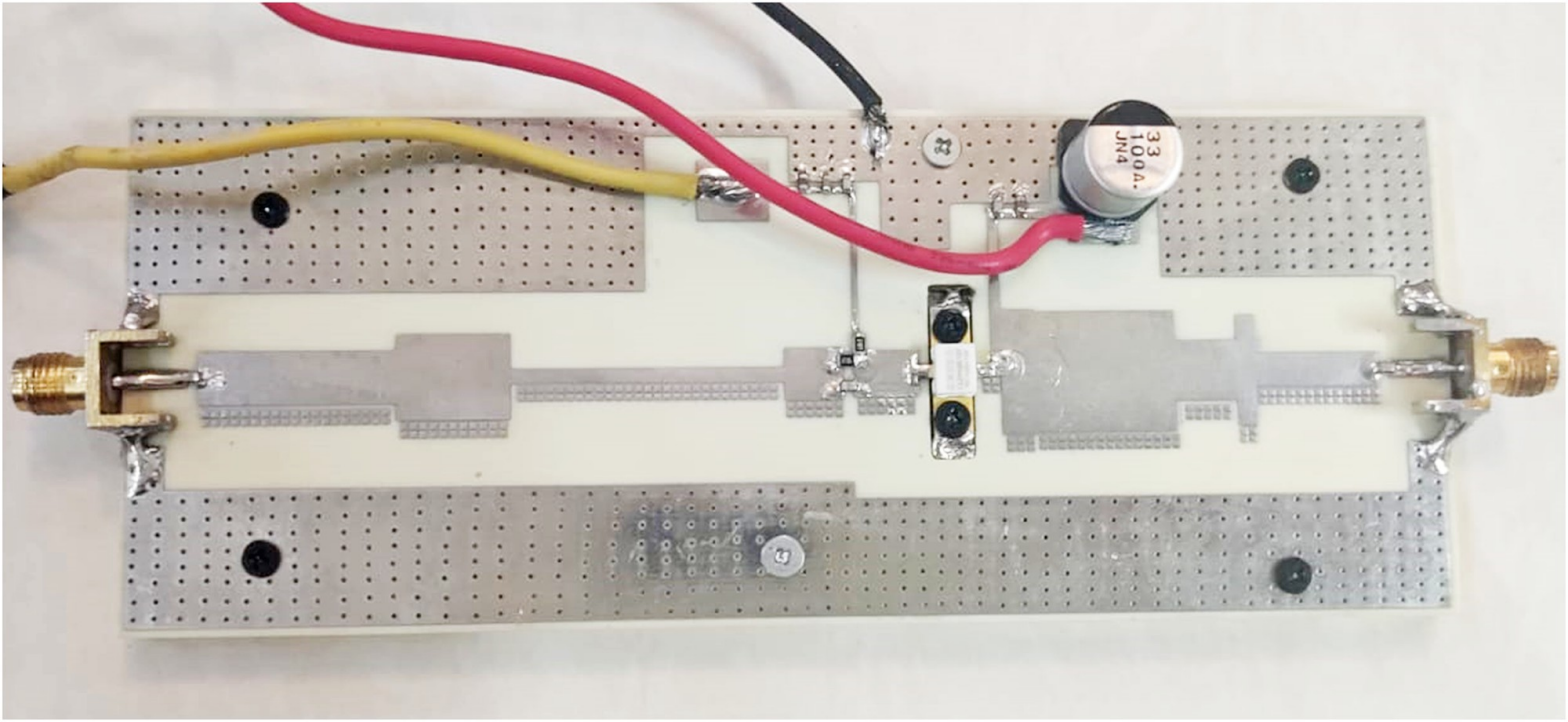
The fabricated power amplifier.

In the proposed circuit in Fig 3, the parallel resistor and capacitor and the two first microstrip lines in the transistor gate that are specified with $\frac{W_{g1}}{L_{g1}}$ and $\frac{W_{g2}}{L_{g2}}$, are used to stabilize the PA. The values of the resistor and capacitor and $\frac{W_{g1}}{L_{g1}}$ and $\frac{W_{g2}}{L_{g2}}$ are selected in such a way as to stabilize the PA at all frequencies. It should be noted that the values of the selected resistor and capacitor and $\frac{W_{g1}}{L_{g1}}$ and $\frac{W_{g2}}{L_{g2}}$ are fixed and don’t vary among the optimization algorithm. After optimizing the PA, the *Mu* factor of the PA was obtained, as shown in Fig 5. For a PA to be stable, the *Mu* factor must be greater than one [19]. The simulation results show that the amplifier is stable at all frequencies. Fig 6 shows the fabricated PA.

**Fig 7:**
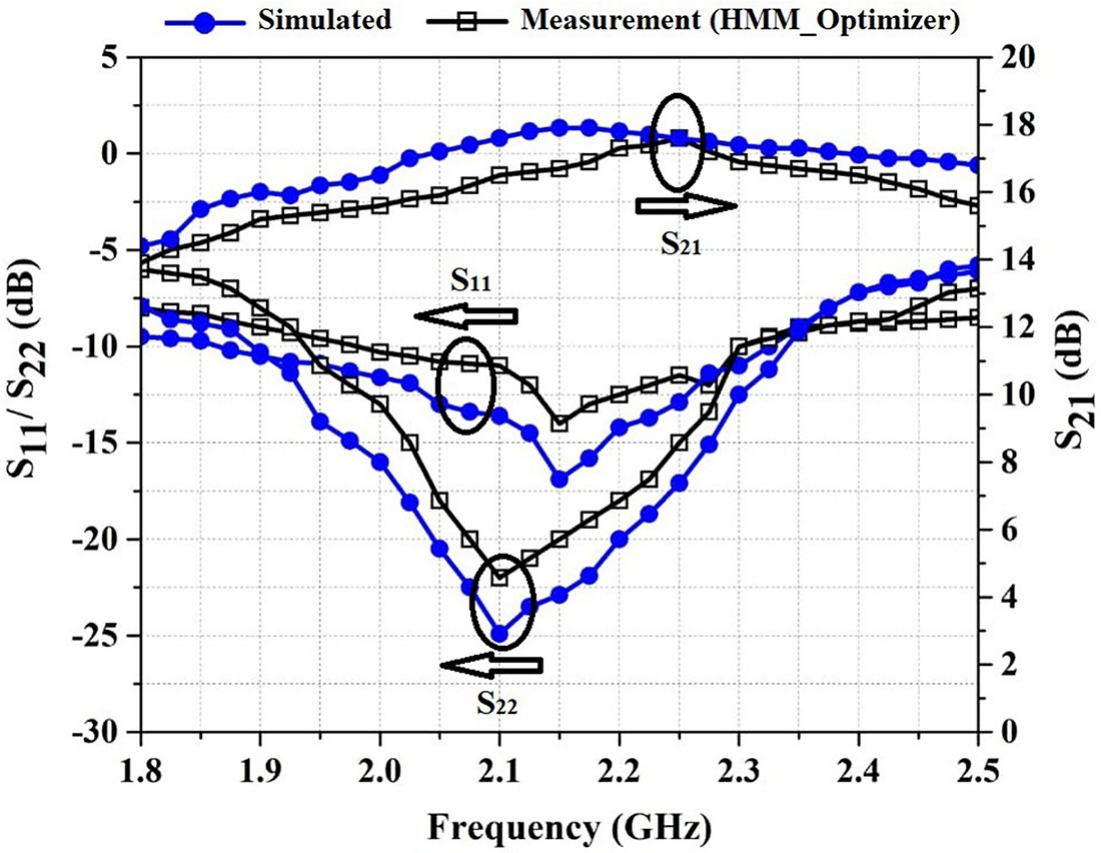
S-parameters versus frequency.

*S*_11_, *S*_22_ and *S*_21_ versus frequency are shown in Fig 7. As is shown in Fig 7, the simulated and measured results are in quite good agreement. The average of the *S*_11_ and *S*_22_ are lower than -9.5 dB and -10.5 dB in the frequency range of 1.8–2.5 GHz, respectively. The values of the *S*_21_ is above 14.5 dB in the frequency range mentioned.

**Fig 8:**
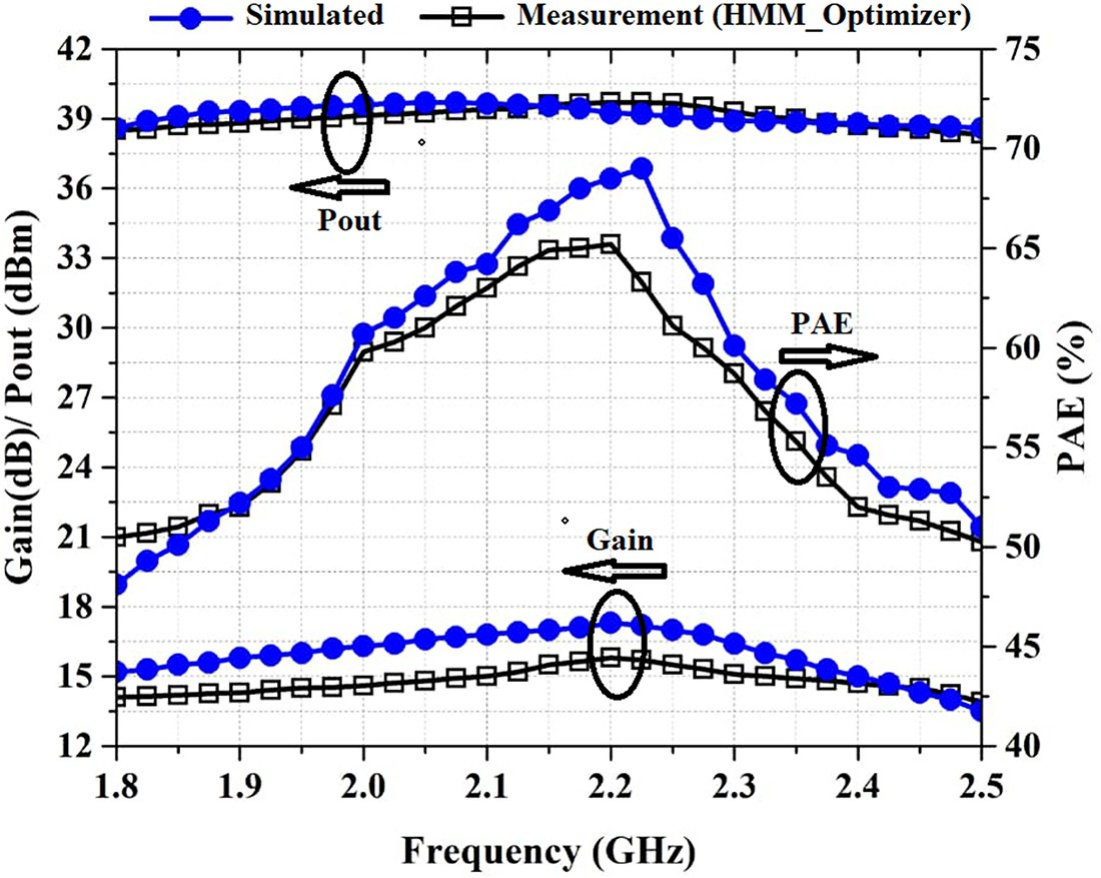
Simulated and measured Gain, P_out_, and PAE versus frequency at P_in_ of 24 dBm.

Fig 8 shows the simulated and measured Pout, PAE, and gain of the proposed PA versus frequency. According to the measurement values specified in Fig 8, PAE is above 50% in the frequency range of 1.8–2.5 GHz and is above 60% in the frequency range of 2–2.3 GHz. The output power is above 38.4 dBm in the frequency range of 1.8–2.5 GHz and is above 39 dBm in the frequency range of 1.95–2.35 GHz. Also, the PA provides an average gain of 14.5 dB in the frequency range of 1.8–2.5 GHz.

**Fig 9:**
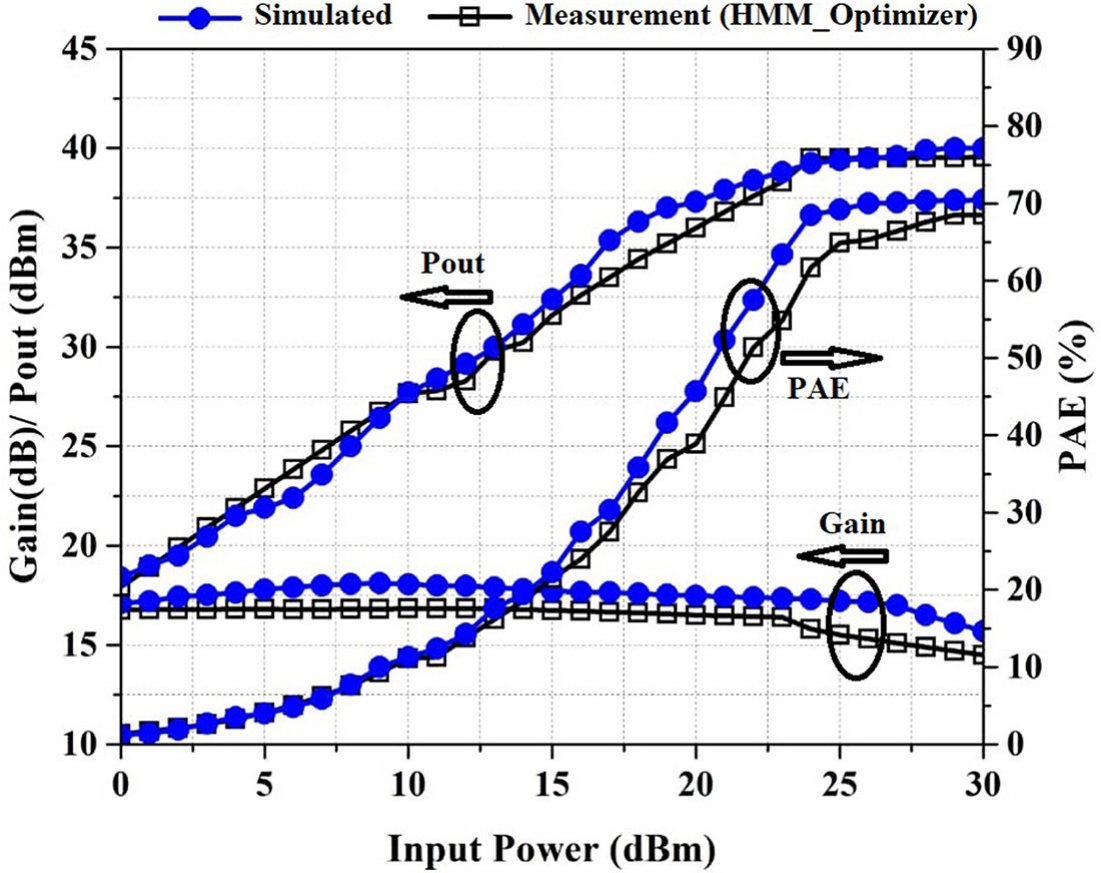
Simulated and measured Gain, P_out_, and PAE versus input power at the frequency of 2.2 GHz.

Pout, PAE, and Gain versus Pin at the frequency of 2.2 GHz are shown in Fig 9. As shown in Fig 9, the fabricated PA provides a PAE above 61.6%, a power gain above the 14.5 dB, and a Pout above 39.5 dBm in the saturation region, where the input power is between 24 dBm and 30 dBm.

**Fig 10:**
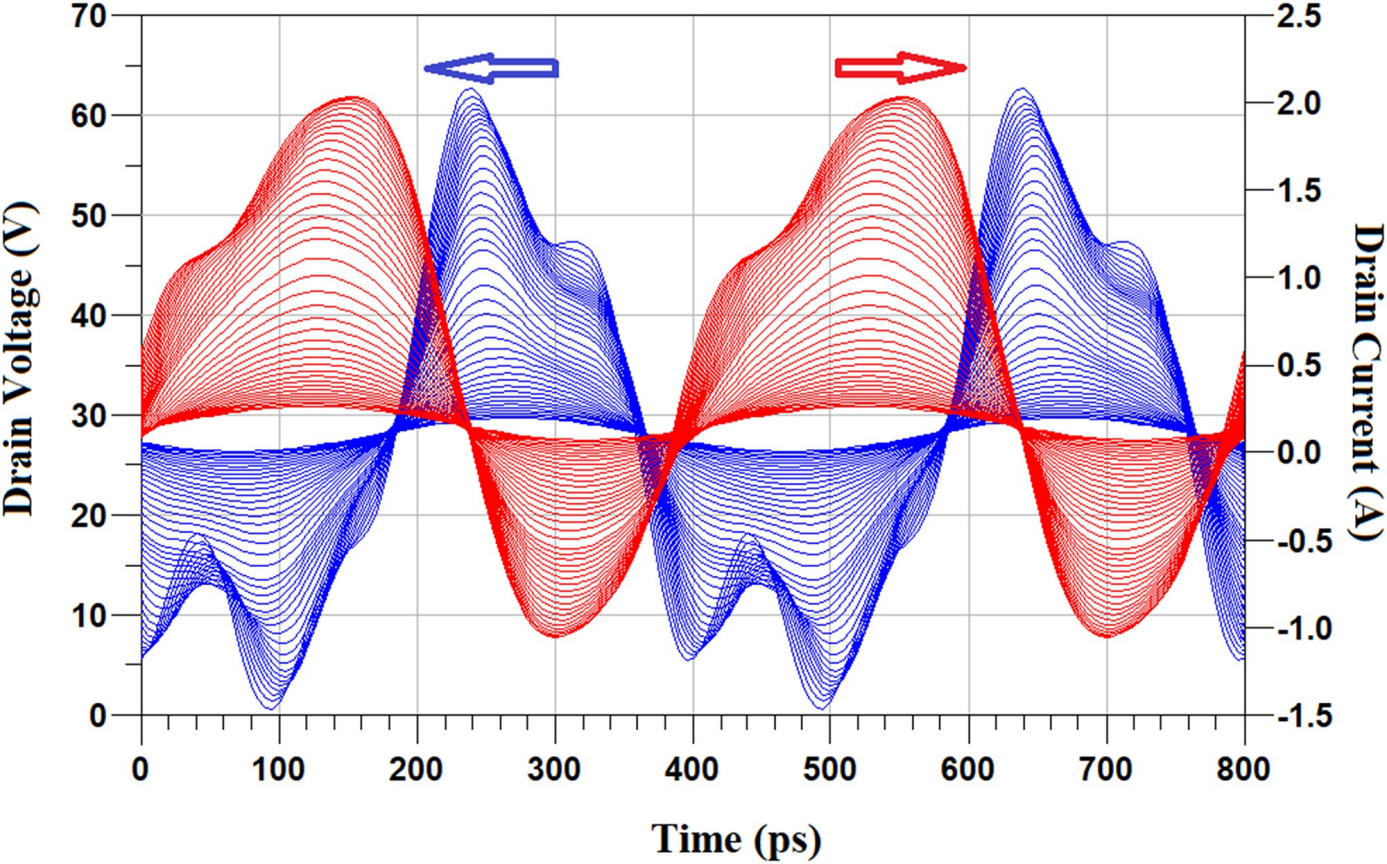
Drain voltage and drain current waveforms of the proposed PA versus P_in_ at the frequency of 2.2 GHz, P_in_ is swept from 0 dBm to 30 dBm.

Fig 10 shows the drain voltage and drain current waveforms of the proposed PA versus Pin at the frequency of 2.2 GHz. Input power, P_in_ is swept from 0 dBm to 30 dBm. When input power increases, the output capacitor enters into its nonlinear region which leads to reducing the phase overlap between the drain current and the drain voltage and so increases the overall PA efficiency.

**Fig 11:**
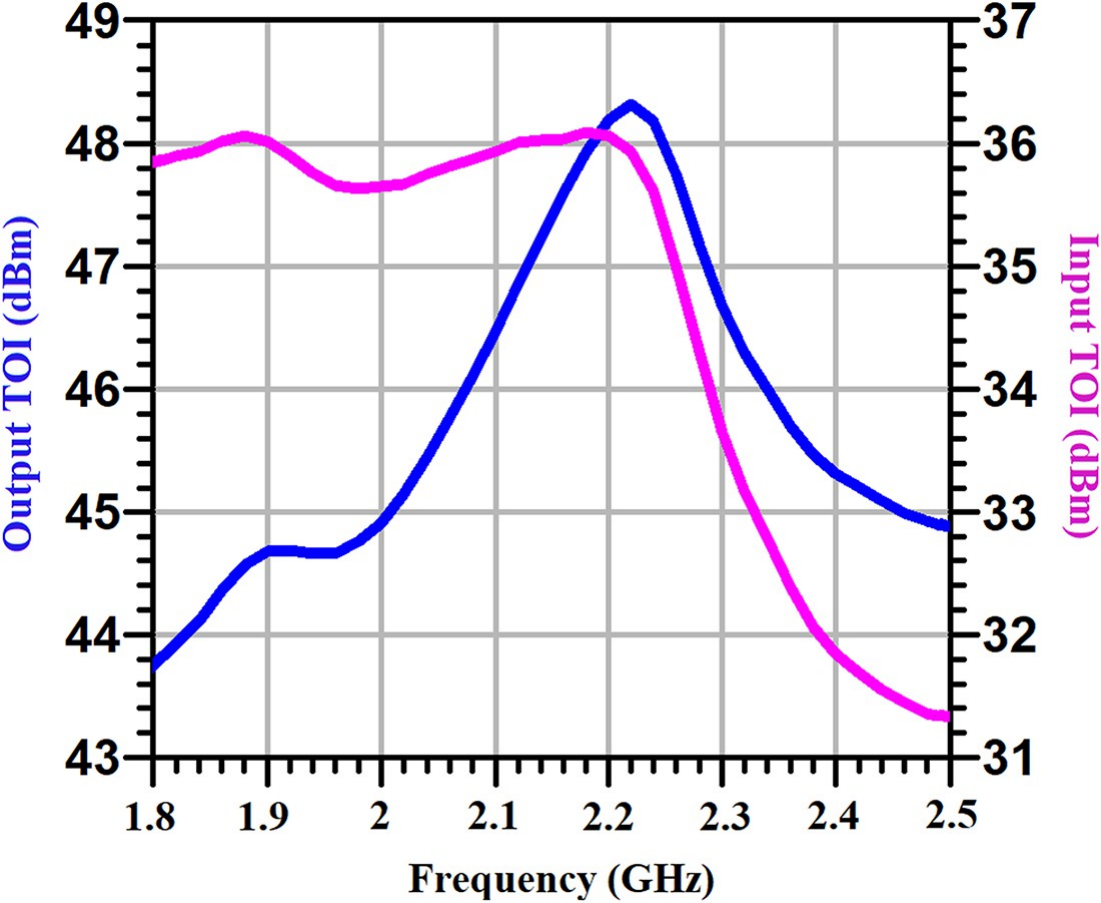
The input and output third-order intercept (TOI) of the proposed PA versus frequency.

Fig 11 shows the input and output third-order intercept (TOI) of the proposed PA versus frequency. The input and output third-order intercept (TOI) are important linearity parameters for PA characterization, as strong narrow-band interferers can exist in the desired bandwidth. As shown in Fig 11, across the bandwidth the input and output TOI of the proposed PA are higher than 31.3 dBm and 43.8 dBm, respectively, where a two-tone test is performed with 1 MHz spacing.

**Fig 12:**
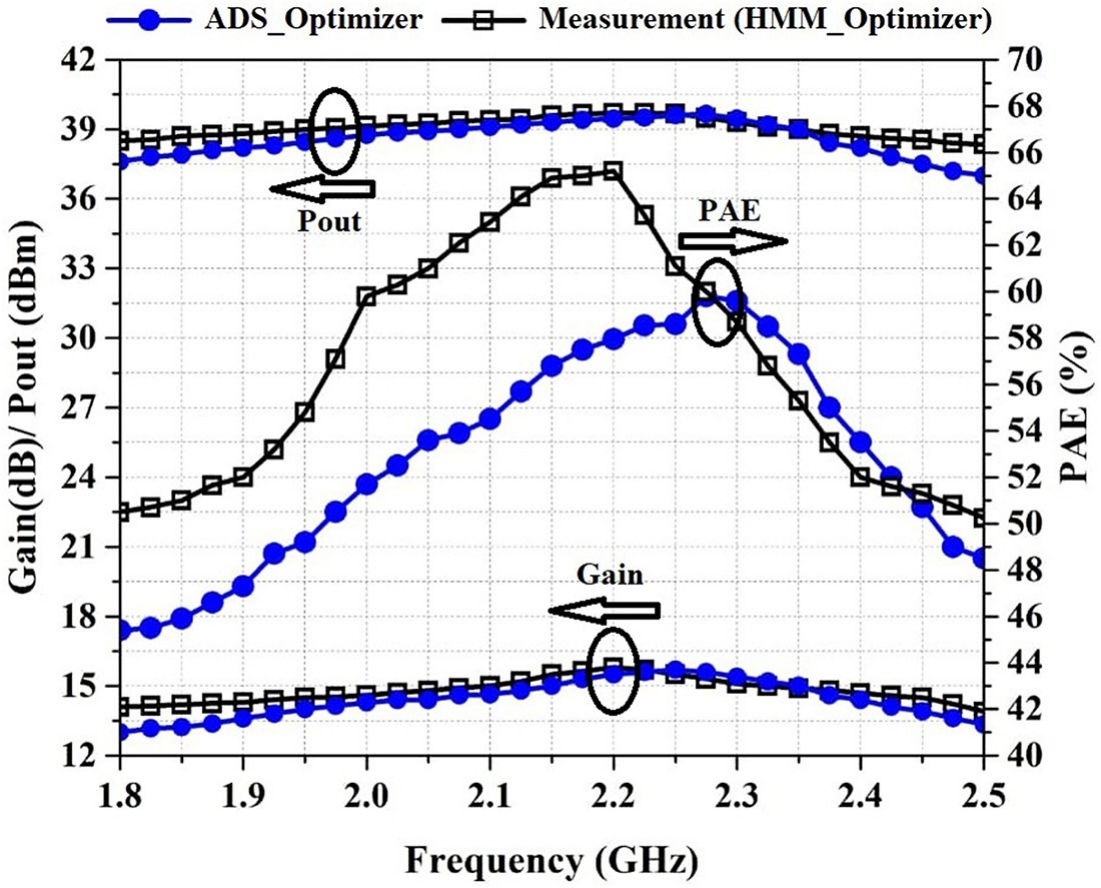
Gain, P_out_, and PAE versus frequency at P_in_ of 24 dBm for PA optimized by ADS_Optimizer and HMM_Optimizer.

**Fig 13:**
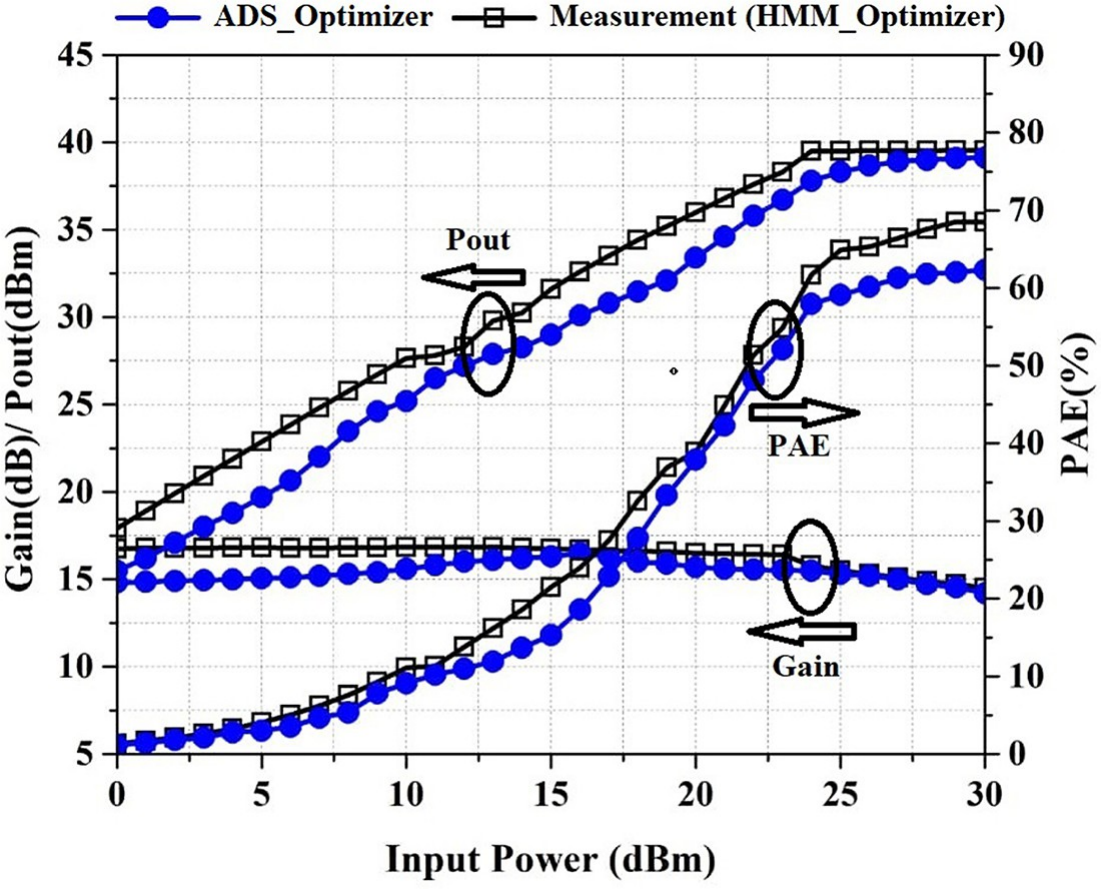
Gain, P_out_, and PAE versus input power at the frequency of 2.2 GHz for PA optimized by ADS_Optimizer and HMM_Optimizer.

For verifying the proposed algorithm, the PA is optimized by the ADS gradient optimizer. P_out_, PAE, and Gain are defined as the main goals. Fig 12 shows the Pout, PAE, and gain of the PA optimized by ADS_Optimizer and HMM_Optimizer versus frequency at P_in_ of 24 dBm and Fig 13 shows these results versus input power at the frequency of 2.2 GHz. By investigating the results that are shown in Figs 12 and 13, we can see that the average of PAE, Pout and Gain obtained by the HMM_Optimizer is higher than the ADS_optimizer. A brief comparison of the two algorithms is summarized in Tables 1 and 2. In Table 2, the comparison is considered in the saturation region, where the input power is between 24dBm and 30dBm.

**Table 1:** A brief comparison between HMM optimizer and ADS optimizer for results obtained by swept of frequency (GHz).

Gain (dB)	PAE (%)	Pout (dBm)	Optimizer Type
14<Gain (1.8 <Ferquency<2.25)	50.5<PAE (1.8 <Ferquency<2.275)	39<Pout (1.95 <Ferquency<2.35)	HMM_optimizer
12.7dB<Gain <15.35dB (1.8 <Ferquency<2.25)	45.4<PAE <59.8 (1.8 <Ferquency<2.275)	38.15 <Pout<39.22 (1.95 <Ferquency<2.35)	ADS_optimizer

**Table 2:** A brief comparison between HMM optimizer and ADS optimizer for results obtained by swept of input power (dBm).

Gain (dB)	PAE (%)	Pout (dBm)	Optimizer Type
14.5<Gain <15.8 (24 <Pin<30)	61.67<PAE <68.5 (24 <Pin<30)	39.5<Pout (24 <Pin<30)	HMM_optimizer
14.21<Gain <15.5 (24 <Pin<30)	57.95<PAE <62.35 (24 <Pin<30)	37.8<Pout (24 <Pin<30)	ADS_optimizer

The obtained results are also compared with some previously published similar works that are shown in Table 3. As shown in Table 3, the fabricated PA has a better performance in comparison with other similar works. This improvement can be seen in the PAE, Gain, and output power of PA.

**Table 3:** Comparison of the proposed PA with some other proposed S-band PAs.

Parameters	Transistor Model	Substrate	BW(GHz)	FBW(%)	Gain (dB)	PAE(%)	V_DD_/I_DSQ_	P_out_ (dBm)	Complexity
Ref.									
[27]	Cree CGH40010F	RO4350B	0.8–3	115	8.3–14.3	55–68 (DE)	28 / 60 mA	41.2 (Average)	Yes
[30]	Ampleon CLF1G0060-10	RO4350B	1.8–2.2	20	10.1–11.6	64.3–79.7 (DE)	50 V/40 mA	14.1–16.6 (W)	No
[40]	Cree CGH40010	N/A	2–4	66	12.3–14.1	36.5–53.4	28 V/ 200 mA	40	No
[41]	Cree CGH40010F	RO4350B	1.9–2.9	42	10–12.5	37–69	28 V/220 mA	36–40	No
[42]	Cree CGH40010	Taconic TLX-8	2–3	66	11.5–12.5	58–72	28/ 60 mA	N/A	Yes
[43]	Cree CGH40010	RO4350B	1.85–2.7	38	10–11.8	68-77(DE)	28 V/155 mA	40.3–41	Yes
[44]	Cree (10 W) N/A	N/A	2.3–2.7	16	12	57–66	N/A	>40	No
[45]	Mitsubishi MGF0840G	RO4350B	1.65–2.75	50	<14	55–72 (DE)	47 V/90 mA	39.5–41.5	Yes
[46]	Cree CGH40010F	RO6035	2.7–3.2	17	10	47	28 V/ 200 mA	<40	No
[47]	Cree CGH40010F	RO4350B	1–2.5	86	12.3–14.1	48–55	28/ 340 mA	N/A	Yes
This work	Cree CGH40010F	RO4003	1.8–2.5	33	13.9–15.8	50.25–65.2	28 V/ 160 mA	38.4–39.7	No

## 5. Conclusion

In this paper, we designed and fabricated an optimized power amplifier with a GaN HEMT for the wireless application that its widths and lengths were predicted and modeled by HMM. For doing this, we defined a new structure of HMM for modeling the PA that consists of several hidden and observable states based on the number of microstrip lines in the input and output matching network. The proposed HMM consisted of 20 hidden states and each hidden state emitted 8 observable states so that their values was close to the initial values obtained from the load-pull analysis and the design of the corresponding matching networks taking into account the initial tunes. The maximum likelihood concept was applied for the training of HMM and the sum of the dissipation power and output powers in the harmonic frequencies was defined as a fitness function. After training HMM, we obtained the optimum values of the widths and lengths of the microstrip lines. With the optimized values, we simulated and realized the PA. Also, In all steps of optimization and simulation of the PA, the precise non-linear model of the transistor was used. Measurement results showed that the PA obtained a PAE higher than 50%, a Gain of about 14 dB, and input and output return losses lower than -10 dB over the frequency range of 1.8–2.5 GHz.
